# What If Root Nodules Are a Guesthouse for a Microbiome? The Case Study of *Acacia longifolia*

**DOI:** 10.3390/biology12091168

**Published:** 2023-08-24

**Authors:** Joana G. Jesus, Cristina Máguas, Ricardo Dias, Mónica Nunes, Pedro Pascoal, Marcelo Pereira, Helena Trindade

**Affiliations:** 1 Centre for Ecology, Evolution and Environmental Change (cE3c), Faculty of Sciences, University of Lisbon (FCUL), Global Change and Sustainability Institute (CHANGE), 1749-016 Lisboa, Portugal; jgjesus@fc.ul.pt (J.G.J.); cmhanson@ciencias.ulisboa.pt (C.M.); rpdias@ciencias.ulisboa.pt (R.D.); 2 Biosystems and Integrative Sciences Institute (BioISI), 1749-016 Lisboa, Portugal; 3 Centro de Testes de Ciências, Faculdade de Ciências da Universidade de Lisboa, 1749-016 Lisboa, Portugal; msnunes@ciencias.ulisboa.pt (M.N.); pfpascoal@ciencias.ulisboa.pt (P.P.); mlpereira@ciencias.ulisboa.pt (M.P.)

**Keywords:** *Bradyrhizobium*, *Coniochaeta*, fire event, histology, infected cells, NGS, starch, symbiosis

## Abstract

**Simple Summary:**

*Acacia longifolia* is an invasive plant highly dispersed in the Mediterranean region. Being a legume, the association with nitrogen-fixing bacteria (i.e., rhizobia) allows access to readily fixed nitrogen, facilitating its growth, development, and dispersal. The extent to which this relation is restricted to bacteria was investigated in this study. We aimed to (i) characterize the microbial community inside root nodules, i.e., structures where nitrogen fixation occurs, and (ii) understand how these partners change following a fire event. In fact, fire potentiates *A. longifolia* invasion, so it is of remarkable interest to understand the role of nodulation in dispersal. We found that root nodules are a guesthouse for microorganisms and, while some are common between unburnt and burnt sites, others shift, particularly considering fungal community.

**Abstract:**

*Acacia longifolia* is one of the most aggressive invaders worldwide whose invasion is potentiated after a fire, a common perturbation in Mediterranean climates. As a legume, this species establishes symbioses with nitrogen-fixing bacteria inside root nodules; however, the overall microbial diversity is still unclear. In this study, we addressed root nodules’ structure and biodiversity through histology and Next-Generation Sequencing, targeting 16S and 25S-28S rDNA genes for bacteria and fungi, respectively. We wanted to evaluate the effect of fire in root nodules from 1-year-old saplings, by comparing unburnt and burnt sites. We found that although having the same general structure, after a fire event, nodules had a higher number of infected cells and greater starch accumulation. Starch accumulated in uninfected cells can be a possible carbon source for the microbiota. Regarding diversity, *Bradyrhizobium* was dominant in both sites (ca. 77%), suggesting it is the preferential partner, followed by *Tardiphaga* (ca. 9%), a non-rhizobial Alphaproteobacteria, and *Synechococcus*, a cyanobacteria (ca. 5%). However, at the burnt site, additional N-fixing bacteria were included in the top 10 genera, highlighting the importance of this process. Major differences were found in the mycobiome, which was diverse in both sites and included genera mostly described as plant endophytes. *Coniochaeta* was dominant in nodules from the burnt site (69%), suggesting its role as a facilitator of symbiotic associations. We highlight the presence of a large bacterial and fungal community in nodules, suggesting nodulation is not restricted to nitrogen fixation. Thus, this microbiome can be involved in facilitating *A. longifolia* invasive success.

## 1. Introduction

Climate change is altering ecosystems’ functioning and services worldwide [1]. Forest ecosystems are particularly threatened by global change, due to the increasing dryness that causes the intensification and magnitude of fire occurrence [2,3]. The effects of fire are broader than vegetation loss, leading to a disruption in soil cycles [4]. This disruption can be easily associated with a loss in microbial diversity, since microorganisms play essential roles, making them essential for soil structure, functioning, and fertility, due to its vital role in nutrient cycling and decomposition [5,6]. The impact of fire on microbial processes is understudied, and the extent to which it compromises biodiversity and species recovery is unclear, albeit fundamental. Fire causes an unbalance at community level, disrupting stability [7], pressuring communities, and making ecosystems vulnerable to invasive species.

Plants are intimately associated with a wide variety of processes, some involving microorganisms [5], and this interaction must be considered for overall ecosystem stability. For example, biological nitrogen (N) fixation contributes to almost half of the N available in terrestrial ecosystems [8,9]. This process involves plants from the Leguminosae family that establish symbiosis with rhizobia, a group currently including Alpha- and Betaproteobacteria from the phylum Pseudomonadota (formerly Proteobacteria) [10], integrated in the large group of Plant Growth Promoting Bacteria (PGPB) [11]. However, diazotrophs (i.e., nitrogen-fixing bacteria) are a broader group that includes archaea, the phylum Actinomycetota (formerly Actinobacteria), and cyanobacteria [12,13]. Generally, the symbiotic process takes place inside root nodules, with nodulation improving plant overall fitness as well as community productivity [14].

Nodule formation is triggered by coordinated signals involving both plant and bacterial factors, leading to a chain of events that includes the infection of root cells, and ending with the development of determinate (i.e., spherical) or indeterminate (i.e., elongated) structures [15,16]. Infection threads (ITs) are formed, allowing bacterial traveling, culminating in successive multiplications until bacteroids’ functional state is achieved, corresponding to the N-fixing form. The nodule develops a vascular system (i.e., vascular bundles), establishing the plant–bacteria connection routes and enabling the exchange of fixed N products loaded in the xylem. In exchange, the plant provides carbohydrates through phloem [17] required for bacterial metabolism [18]. Apart from this vascular system (outer cortex), nodules have a layer of cells (inner cortex) in which microaerophilic conditions are created due to leghemoglobin production [19], allowing nitrogenase (i.e., the enzyme that performs N fixation) to function [20]. Nodulation is a highly regulated process that is considered an important adaptative trait for plants, especially under oligotrophic conditions. It plays a determinant role in ecosystems, and legumes are known to be ecosystem-pioneers [21]. However, in spite of the highly regulated genetic feedback that disentangles nodulation and triggers a specific association, i.e., one host and one symbiont, the nodule environment seems to be more embracing. As noted by Etesami [22], nodules may be colonized by non-rhizobial bacteria, extending the concept of nodulation beyond N fixation. These partners can contribute with plant growth-promoting (PGP) factors, improving plant fitness and behavior. Both culture-dependent [23,24] and culture-independent methods [25] provide support for this wider diversity, including the class Gammaproteobacteria and the phylum Bacillota (formerly Firmicutes). For this reason, host promiscuity is being considered as an important characteristic for invasive plants regarding the establishment in novel ecosystems.

Our group has been studying *Acacia longifolia* (Andrews) Willd. which is now classified as one of most successful invasive plants worldwide [26,27]. Native of southern Australia, this species is being widely dispersed in the Mediterranean regions including Portugal, as well as California, and South Africa. As a fast-growing species and with a great accumulation of biomass (e.g., litter), this species is responsible for profound changes in the above- and belowground environments [28], making it a suitable model for studying adaptations. Furthermore, *A. longifolia* forms a massive soil seed bank that is highly stimulated by fire [29], making post-fire conditions an opportunity to disperse, especially if novel and advantageous symbiotic associations can be established [30]. Nodulation and mutualisms, mostly with *Bradyrhizobium* and other Alphaproteobacteria members as symbiotic partners, do not constrain *Acacia* spp. invasion [31]. However, the so-called promiscuity associated with acacias sensu lato, e.g., Rodríguez-Echeverría et al. ref. [32], raises questions about the extent to which bacterial partners are restricted in *A. longifolia*. Jesus et al. [30] showed a broader cultivable bacterial diversity associated with root nodules, aside from the recently included Rhizobia group. Additionally, acacias can also harbor mycorrhizal fungi, both arbuscular and ectomycorrhizal, with the former being predominant in both native and invasive ranges [33,34]. *Acacia longifolia* is a host for fungal species present in native communities [35], having a common mycorrhizal network, and raising the hypothesis of a tripartite relationship (plant–bacteria–fungi). Furthermore, knowing that nodulation potentiates invasion [36], the possibility of adjusting the microbial community that could be harbored inside root nodules could be an important tool for the *A. longifolia* adaptation process, triggering successful dispersal events, as already mentioned by Rodríguez-Echeverría et al. [37]. Furthermore, it is already known that *Acacia* spp. could select a specific bacterial community in its rhizosphere over geographic ranges [38], reinforcing this genus effect in surrounding soil. In this sense, understanding the interaction between those micro-communities and invasive plant species is of upmost importance, especially after fire, when *A. longifolia* post-fire recruitment is highly efficient.

With this study, we aimed to compare the effect of fire on young plants’ nodules (one year after fire). Two different conditions, unburnt and burnt forest ecosystems, were considered, and we explored the internal structure of root nodules through histological techniques, together with microbial profiling based on Next-Generation Sequencing (NGS). We assessed if (i) nodules formed in these two different conditions had a similar structure, (ii) the bacterial diversity harbored inside, and (iii) the fungal diversity and composition. This study provides new knowledge to understand the contribution of the microbiome associated with nodulation to the success of invasive acacia species considering the possible impacts of fire events.

## 2. Material and Methods

### 2.1. Study Site

This research was carried out in Mira, Aveiro, Northern Coastal Portugal (40.52451° N, 8.67253° W) in a mixed forest dominated by Maritime Pine (*Pinus pinaster* Aiton) and *Eucalyptus globulus* Labill., invaded by *Acacia longifolia*. This area is classified as Csb (warm-summer Mediterranean climate) according to the Kӧppen–Geiger classification [39], and was affected by the wildfire of 15 October 2017. Two different conditions were studied, unburnt (hereafter UBS) and burnt (BS) sites, and sampling was performed one year after the fire (in October 2018) by establishing three plots in each site. Root nodules were collected by digging up *A. longifolia* saplings (between 20–80 cm in height), comprising a total of 48 plants collected (8 in each plot) (Figure S1). Furthermore, while some nodules were stored in silica gel and kept at room temperature until further procedures, others were briefly inspected for activity based on the color of the internal tissue. Only the reddish ones were chosen for the fixation protocol as an indication of the presence of leghemoglobin, which is an indirect confirmation of nodule functionality (in accordance with [40]).

### 2.2. Histological Studies

Ten *A. longifolia* root nodules were observed from each site (UBS and BS), selecting the elongated ones with similar size (to capture a similar development stage). Pre-selected nodules (as mentioned in the Section 2.1) were fixed in a 1:1 glutaraldehyde (1.5%) and paraformaldehyde (1.5%) solution before embedding in paraplast. A semi-automatic rotary microtome RM2155 (Leica Biosystems) was used for serial sectioning of longitudinal semi-thin sections (12 µm). Sections were stained with different dyes: toluidine blue (for general observation), cotton blue in lactophenol and Löffler solution (for bacteria observation), and Lugol solution (for starch staining). The images were captured using an Olympus BX51 Brightfield microscope equipped with a TIS DFK 1.9MP Sony CCD color camera and processed in Image J2 (version 1.53t 2022).

### 2.3. Microbial Diversity through Next-Generation Sequencing

Bacterial and fungal profiling was performed to assess diversity inside root nodules. Ten nodules from each sampling site (i.e., three unburnt and three burnt) were selected and treated as a composite sample (UBS vs. BS). After hydration, to ensure nodule outer-surface disinfection, nodules were washed in 10 mL of Washing Solution (PBS + 2 mL/L Tween 20), placed on an orbital shaker at 140 rpm for 3 h, and ultrasound treated using 30 s impulses three times. DNA extraction was carried out using a CTAB-based DNA extraction protocol [41]. The quantification of DNA was performed using the Qubit dsDNA high-sensitivity assay (v 1.01, ThermoFisher Scientific, Lisbon, Portugal), and all nodule DNA samples showed a concentration above 50 ng/μL. Additionally, the purity of the DNA samples was assessed using the Nanodrop™ 2000 (ThermoFisher), with all DNA samples exhibiting purity values between 1.7 and 2.0 for the 260/280 nm and 260/230 nm ratios.

For the rDNA gene amplification, Long Amp hot start Taq 2× master mix (New England Biolabs, MA, USA) was used at 1X along with 50 ng/µL of genomic DNA from *Acacia* nodules. To amplify the full-length 16S rDNA bacterial gene, 0.25 µM of the primer pair 27F (5′-AGAGTTTGATCMTGGCTCAG-3′) and 1492R (5′-CGGTTACCTTGTTACGACTT-3′) were used, while to amplify the 1400 bp length of the Fungal Nuclear Large Subunit (LSU rDNA gene) (i.e., 25S-28S rRNA) region, 0.4 µM of the primer pair RL0R Fw (5′-ACCCGCTGAACTTAAGC-3′) and LR7 (5′-TACTACCACCAAGATCT-3′) were used. Both PCRs were conducted on a Biometra UNO II, using the following conditions for 16S rDNA gene: 1 cycle of 94 °C for 1 min, 35 cycles of 94 °C for 20 s, 55 °C for 30 s, and 65 °C for 2 min, and a final extension of 65 °C for 5 min; and for the 25S-28S rDNA gene: 1 cycle of 95 °C for 3 min, 35 cycles of 94 °C for 30 s, 52 °C for 30 s, and 65 °C for 1 min, and a final extension of 65 °C for 5 min. Subsequently, amplification products were visualized via gel electrophoresis and purified using the Solid Phase Reversible Immobilization (SPRI) technique with magnetic beads [42,43].

Quantification steps were performed using the 1xdsDNA HS assay for Qubit. DNA was end-repair with the NEBNext^®^ End Repair Module (New England BioLabs, Ipswich, MA, USA), cleaned with Agencourt AMPure XP Beads (Beckman Coulter, High Wycombe, UK) and dA-tailed (New England BioLabs, Ipswich, MA, USA). The library was prepared from 300 ng input DNA from each sample using the Sequencing Native Barcoding Kit 24 V14 (SQK-NBD114.24) (Oxford Nanopore Technologies, Oxford, UK) in accordance with the manufacturer’s protocol. The library was quantified and prepared for PromethION sequencing, using FLO-PRO114M flowcells, MinKNOW v22.12.4, standard 72 h run script with active channel selection enabled. A period of 24 h yielded 3,000,000 passed reads with an estimated N50 of 1500 bp and the mean quality score was 14. In total, 4.5 Gb of data were produced, with an average of 750,000 reads per sample.

### 2.4. Data Analysis

Images from histological observations were processed in Image J2(version 1.53t 2022. This software was also used to compare the quantity of infected cells inside root nodules’ cells from each condition (UBS vs. BS) (according to Collins [44]).

Sequencing data were carefully processed and analyzed through a series of critical steps to ensure the accuracy and reliability of the results, namely:

Preprocessing—Filtering for Size and Quality:

After the removal of low-quality reads, the remaining reads were further filtered using Prinseq-lite version 0.20.4 [45]. The filtering process was based on size and quality criteria. Specifically, reads with lengths longer than 1200 base pairs (bps) and shorter than 1700 bps were retained for subsequent analysis. Additionally, reads with a minimum Phred score of 7 were considered acceptable. This rigorous filtering ensured that only high-quality and informative reads were used in downstream analyses.

Taxonomic Classification:

The taxonomic classification of the preprocessed reads was performed using Kraken 2 (version 2.1.2). Kraken 2 is a powerful bioinformatic tool known for its fast and accurate taxonomic classification of DNA sequences. It employs a Lowest Common Ancestor (LCA) approach, where k-mers (short subsequences of DNA) from the input reads are mapped to the lowest common ancestor shared by all known genomes containing that k-mer [46]. This classification method aids in identifying the taxonomic origin of the reads, providing valuable insights into the microbial composition of the samples.

In this classification, a comprehensive reference database was used, which included the NCBI RefSeq reference genomes and NCBI GenBank reference sequences of Archaea, Bacteria (up to May 2023), and Fungi (up to April 2022). By comparing the preprocessed reads against this extensive collection of known genomes, Kraken 2 accurately assigned taxonomic classifications to the reads, contributing to a thorough understanding of the microbial communities present in the acacia nodules samples. When reads were considered unclassified, it means that the database lacked the required level of detail to make an accurate classification to specific taxonomic levels, leading to “unclassified” assignments. This limitation hinders the precise identification of certain organisms or taxa within the dataset used.

Using the operational taxonomic units (OTUs) for the three plots within each site (UBS and BS), Shannon–Wiener diversity and Pielou evenness were calculated. For Bray–Curtis dissimilarity, a mean value for each site was compared to infer dissimilarity between bacteria and fungi present. These indexes were calculated using the packages vegan [47] in R studio (v.2023.03.0).

## 3. Results

### 3.1. Histological Studies

**Figure 1:**
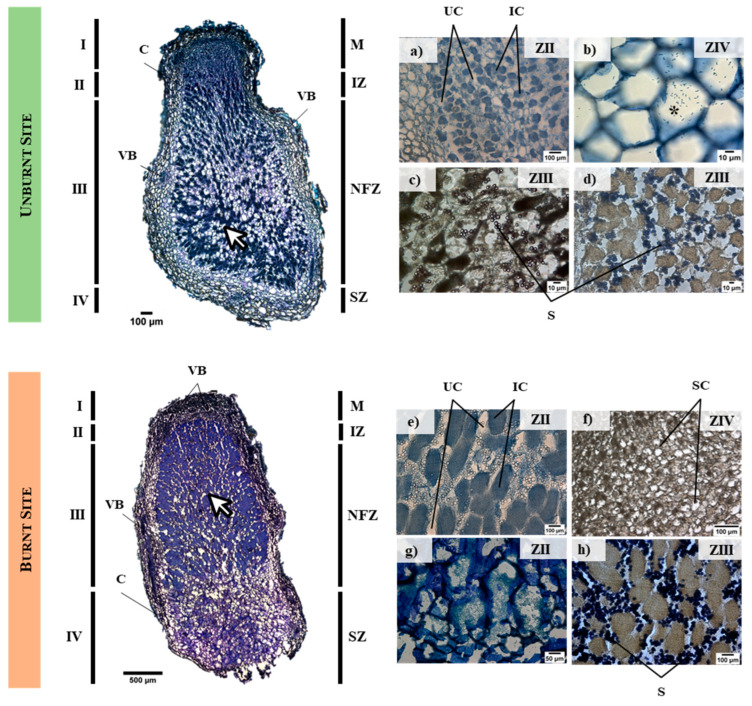
Longitudinal semi-thin sections (12 µm) of *Acacia longifolia* root nodules collected in unburnt and burnt sites. Nodules have four different zones: meristematic zone with a persistent meristem (M) or zone I, infection zone (IZ) or zone II, N-fixing zone (NFZ) or zone III, and senescence zone (SZ) or zone IV. Vascular bundles (VB) are present in the nodule cortex (C). The central infected zone contains infected (IC) and uninfected (UC) cells, and starch granules (S) are present in UC in close vicinity to IC. Senescent cells (SC) were observed in SZ. Reconstructed images on the left were stained with toluidine blue. Magnified images on the right side refer to three different insets: infection zone (identified as ZII), nitrogen-fixing zone (ZIII), and senescent zone (ZIV). Insets (**a**,**b**,**e**) are stained with cotton blue in lactophenol, (**c**,**f**) are unstained, (**d**,**h**) are stained with Lugol solution, and (**g**) is stained with Löffler solution. (*) represents rod-shape bacteria inside IC and arrows highlight the quantity of IC. Note that scale bars are different and adjusted to each image.

Root nodules developed in unburnt and burnt sites revealed a similar internal organization, as revealed by longitudinal semi-thin sections (Figure 1, left). Four different zones were found, namely, meristematic (M or I), infection (IZ or II), nitrogen-fixing (NFZ or III), and senescence zones (SZ or IV). Our observations revealed root nodules were highly vascularized, with vascular bundles (identified as VB) around the nodule cortex (marked as C). Furthermore, our repeated observations showed differences in the nodule internal structure, the most remarkable being the quantity of infected cells (IC; arrows on Figure 1) (i.e., cells infected by bacteria) in the two studied sites, with a higher quantity in nodules from the burnt site compared with unburnt one (80% and 64%, respectively). These IC were present within zones II and III, and were more abundant in zone III (i.e., NFZ), decreasing towards the senescence zone (Figure 1, right—insets on ZIV), the zone closest to the plant root. Lugol staining revealed spherical starch granules accumulated in uninfected cells (UC) near to infected ones, and decreasing towards SZ. Furthermore, a pattern was observed in which more IC resulted in the accumulation of more starch granules, particularly in NFZ (insets on ZIII). Senescent cells (marked as SC) were mostly free of bacteria, with broken cell walls and without starch.

### 3.2. Microbial Community Composition and Diversity inside Root Nodules

Sequencing recovered a total of 799,338 classified reads for bacteria through 16S rRNA identification (17% remained as unclassified) distributed in 486 ± 90 and 453 ± 54 operational taxonomic units (OTUs) for the unburnt and burnt sites, respectively (Table 1). Considering sequencing targeted for fungi, a total of 2,030,293 classified reads were distributed in 705 ± 133 and 531 ± 93 OTUs for unburnt and burnt sites, respectively (18% remained as unclassified). Owing to the high depth of sequencing, the rarefaction curves showed a high coverage approaching saturation in all the samples for both bacteria and fungi (Figure S2). Furthermore, along with bacterial sequencing, we found a residual representation of archaea with 18 ± 9 and 5 ± 4 OTUs for unburnt and burnt sites, respectively. In addition, considering the Shannon–Wiener diversity index, we found similar values for unburnt and burnt sites considering bacteria (4.15 ± 0.354 and 4.20 ± 0.187, respectively), while a clear decrease was found for fungal diversity in the burnt site (2.50 ± 0.656 compared with 3.66 ± 0.186 for the unburnt site). The Pielou evenness index showed a higher evenness for bacterial communities compared with fungal, with a dominance evident in the burnt site with the lowest value (0.363 ± 0.09). Considering beta diversity, Bray–Curtis dissimilarity showed higher dissimilarity in the fungal rather than the bacterial community (0.61 and 0.25, respectively), when comparing the two sites (Table 1, Figure S3).

**Figure 2:**
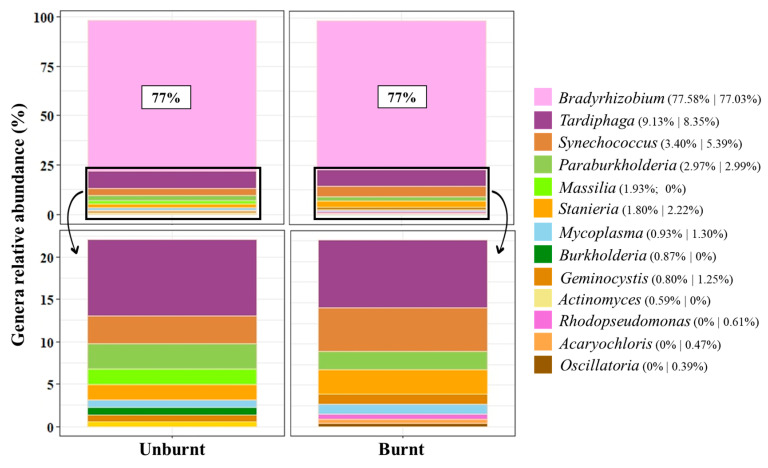
Top 10 genera of the bacteria identified in *Acacia longifolia* root nodules (cumulative relative abundance, %), for unburnt and burnt sites. The bottom graph represents an inset of the abundance excluding *Bradyrhizobium* (23%). The percentage was calculated based on the mean value of reads assessed for each site (*n* = 3) and is presented in the legend considering unburnt followed by burnt values. Colors are assigned according to bacterial classes (gradient of purple, Alphaproteobacteria; gradient of green, Betaproteobacteria; gradient of orange for Cyanophyceae; blue for Mollicutes).

Regarding bacterial diversity inside *A. longifolia* root nodules, we found a dominance of the phylum Pseudomonadota, followed by Cyanobacteria, Actinomycetota, and Bacillota. The list of the top 10 genera was led by *Bradyrhizobium* (making up 77% for both sites). This genus was followed by *Tardiphaga* (9 and 8%) and *Synechococcus* (3 and 5%), which were the top three genera in unburnt and burnt sites, respectively. Other common genera were found between sites, varying in abundance, namely, *Geminocystis*, *Mycoplasma*, *Paraburkholderia*, and *Stanieria*. Differences were, however, present: for the unburnt site, *Actinomyces*, *Burkholderia*, and *Massilia* were identified, while *Acaryochloris, Oscillatoria*, and *Rhodopseudomonas* were identified in the burnt zone (Figure 2).

**Figure 3:**
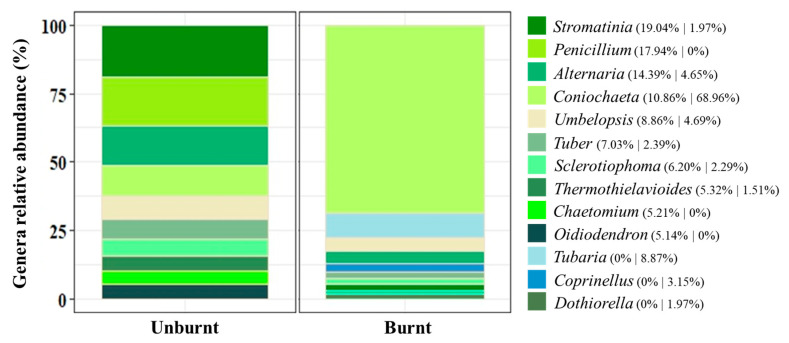
Top 10 genera of the fungal community identified in *Acacia longifolia* root nodules (cumulative relative abundance, %), for unburnt and burnt sites. The percentage was calculated based on the mean value of reads assessed for each site (*n* = 3) and are presented in the legend considering unburnt followed by burnt values. Colors are assigned according to fungal phyla (gradient of green, Ascomycota; gradient of blue, Basidiomycota; grey for Mucoromycota).

Regarding fungal diversity, in the unburnt site, 91% of genera were found to belong to Ascomycota, with 9% remaining for Mucoromycota. In the burnt site, 83% of the genera found were Ascomycota, with Basidiomycota and Mucoromycota accounting for 12 and 5%, respectively. As shown in Figure 3, in the unburnt site we found *Stromatinia* (19%), *Penicillium* (18%), *Alternaria* (14%), and *Coniochaeta* (11%) as the main genera, followed by *Umbelopsis*, *Tuber*, *Sclerotiophoma*, *Thermothielavioides*, *Chaetomium*, and *Oidiodendron*, ranging from 9 to 5% of relative abundance. For the burnt site, there was a dominance of *Coniochaeta* accounting for 69% (also shown in Table 1 with 0.363 ± 0.09 for the Pielou index), followed by *Tubaria*, *Umbelopsis*, *Alternaria*, *Coprinellus*, *Tuber*, *Sclerotiophoma*, *Stromatinia*, *Dothiorella*, and *Thermothielavioides*, sharing almost equally the remaining 31%. Both sites shared 54% of the genera, considering the top 10 rated taxa.

## 4. Discussion

*Acacia longifolia* is regarded as a model species for studying symbiosis in the context of invasion biology, considering its ease in finding suitable partners to establish this interaction and its worldwide distribution. Because symbiosis occurs in root nodules, it is important to study the structure and microbial diversity of these plant organs. To the best of our knowledge, this is the first report in which such a diverse microbiome (including both bacteria and fungi) is described for the nodules’ community, suggesting nodulation can be a potential multifunctional trait determinant for *A. longifolia* establishment, development, and success in dispersal.

### 4.1. Fire Boosts Infected Cells and Starch Accumulation

*Acacia* spp. root nodules’ structure and functionality were described by Sprent et al. [48]. Accordingly, and as a legume from a temperate region, *A. longifolia* develops indeterminate-type nodules (i.e., elongated). This was confirmed by our observations, which means that a persistently active meristem is present allowing the elongation of nodule tissues, e.g., [49,50,51].

Furthermore, our observations are in accordance with Vasse et al. [52], who described the same varied histological zones for the model legume *Medicago sativa* L. This differentiation occurs due to the continuous development stages until the root nodule is formed: meristematic zone (I) corresponding to the active growth zone followed by infection zone (II), where nodule infection occurs via IT. Zone III is the N-fixing zone where bacteria are already differentiated into bacteroids able to fix N. Lastly, the senescence zone (IV) is attached to the root, and is where cells are generally degraded and bacteria are no longer functional [53,54,55]. Furthermore, we also found that *A. longifolia* root nodules are highly vascularized (see Figure 1, VB), being efficient at maintaining water and exchanging organic compounds and symbiotic products, e.g., [56].

After fire occurrence, considering internal anatomy, there was an increase in the number of infected cells (IC), suggesting that infection is potentiated (white arrows in Figure 1). Since fire is a disturbance that enhances *A. longifolia* seed germination and invasion success [29], this increased microbial proliferation suggests that nodulation is a process contributing to plant development, facilitating establishment. In addition, a higher infection corresponds to a higher bacterial load (i.e., 80% of IC compared with 64% for the unburnt site). Furthermore, we observed that starch granules accumulated in uninfected cells (Figure 1b, iv, inset on ZIII), corroborating the findings described by Newcomb et al. [57] in *Pisum sativum* L., who noted spherical starch granules within uninfected cells (UC). We also found a relation between IC and starch, with more IC followed by more starch, suggesting that starch is available to be metabolized as a source of carbon (C) for bacteria development. Indeed, this was shown by other studies that found an accumulation of starch in the early stages of root nodule development, allowing it to be metabolized by bacteroids when root nodules are actively fixing N [58]. Following this evidence, we also found that, in the senescence zone (see Figure 1, SZ), i.e., in which bacteria are already inactive, starch was also absent.

### 4.2. Acacia longifolia Harbours a Microbiome inside Root Nodules

The root nodule bacteriome presented differences when comparing unburnt and burnt sites. As previously described for invasive and native ranges, the dominant bacterial partner is *Bradyrhizobium*, including a range of species within this genus [30,59,60,61], reinforcing its role as a preferential partner for N fixation. *Tardiphaga* and *Synechococcus* were the second and third most abundant genera, maintaining its relative abundance between sites. *Tardiphaga* is an Alphaproteobacteria, which is a non-rhizobial genus already identified inside *Glycine max* (soybean) root nodules [22,24], and carrying N-fixing genes [62]. Interestingly, while several species of *Bradyrhizobium* were recruited by *A. longifolia*, only one species from *Tardiphaga* (i.e., *Tardiphaga robiniae*) and *Synechococcus* (*Synechococcus* sp. JA-3-3Ab) were identified inside root nodules, highlighting the ease of recruiting and preference for *Bradyrhizobium* spp.

Cyanobacteria were described here for the first time inside root nodules; however, a mutualistic interaction between cyanobacteria and angiosperms was first reported by Osborne et al. [63]. Their presence highlights the broader distribution and ecological plasticity of this phylum [64]. *Synechococcus* was the most abundant, followed by *Geminocystis* and *Stanieria* in unburnt and burnt sites, while *Acaryochloris* and *Oscillatoria* dominated at the burnt site. Recent studies reported that cyanobacteria, as mixotrophs, may survive using non-photosynthesized C [65], generally provided by heterotrophic bacteria [66,67]. This ability allows cyanobacteria to successfully colonize other environments, including soils, opening the possibility to live inside root nodules. In addition, due to the close phylogenetic relationship with Alphaproteobacteria (i.e., rhizobia) [13], cyanobacteria can be easily recruited into *A. longifolia* root nodules where they can contribute to N fixation [66,68,69,70], reinforcing its entrance and possible function.

*Massilia* and *Burkholderia* have been associated with plant growth, development and defense, tissue differentiation, and cell division [22,71,72]. Its higher abundance at the unburnt site suggests *A. longifolia* is recruiting partners for those functions. *Actinomyces* has been reported to occur in soils (revised by Boukhatem et al. [73]), but has not been associated with plants, and its presence inside nodules is reported here for the first time. This genus is also responsible for the production of gibberellin, a phytohormone important for growth [74]. This bacteriome can confer an advantage to *A. longifolia* in an interspecific competitive environment, focusing on its development. On the other hand, in the burnt site, a functional specialization seems to be underway, with the higher presence of *Rhodopseudomonas*, *Acaryochloris*, and *Oscillatoria* associated with N fixation [66,70,75], highlighting the importance of this process to acacia’s post-fire establishment and recovery.

Regarding the *A. longifolia* nodule mycobiome, it is described here for the first time, accounting for more OTUs than total bacteria. The root nodule cortex could be a suitable microhabitat to be colonized by fungi, as shown by the diverse community found in the present study. Fungi take advantage of the fixed N while supplying phosphorus for the other partners, leading to a more effective N-fixing symbiosis [76,77]. We found a dominance of the phylum Ascomycota, previously reported as playing an important role in nutrient cycling and plant interactions [78]. Indeed, the evidence for a tripartite plant–rhizobia–fungi symbiosis inside *A. longifolia* root nodules is being shown here for the first time, although it had been previously described in the rhizosphere [79].

Most of the fungal genera found have been identified previously in various plant organs (including leaves, roots, seeds, and shoots) and considered endophytes, including *Alternaria*, *Chaetomium*, *Coniochaeta*, *Coprinellus*, *Dothiorella*, *Oidiodendron*, *Penicillium*, *Tuber*, and *Umbelopsis* [80]. This may explain why most of them (ca. 54%) were found at both unburnt and burnt sites. Indeed, whether these fungi are endotrophs or true endophytes remains to be clarified, as well as the hypothesis of vertical transmission to the offspring via seeds of these endotrophs [81]. In the context of fire, their presence can be advantageous for *A. longifolia* post-fire germination and establishment. Moreover, plant–microbe associations, as endophytes, can be highly determinant in plant defense [82], nutrient acquisition [83], and abiotic stress resistance [84], enhancing plant fitness.

Post-fire regeneration could be enhanced by microbial communities with fungi suppling nutrients and bacteria transforming C and N [85]. Furthermore, the interaction between microbial groups (bacteria and fungi) seems also to be relevant. At the burnt site, there was a clear dominance of *Coniochaeta*, a genus belonging to the phylum Ascomycota, which interacts with plants by secreting proteins that trigger symbiotic associations [78]. Since, after fire, *A. longifolia* germination is potentiated, *Coniochaeta* could be a facilitator for reestablishment of nodulation. As mentioned, most of the genera found are described as endotrophs or true endophytes; however, a gap in knowledge remains regarding the role of each fungal genus, restricting the discussion to the evidence of their presence. However, it was shown for other *Acacia* spp. that acacias could shape their rhizospheric environment [38], which explains the fact that some genera remain within the symbiotic root nodule environment, even after a fire event. Future studies should be focused on metagenomics and transcriptomics of the main genera identified, to address the role they play in nodulation, along with a screening of rhizospheric microbial communities. This will provide further insights into the hypothesis of root nodules as multifunctional structures that enhance *A. longifolia* success.

After fire, several microbial-mediated processes are disrupted [86,87] and a decrease in microbial diversity occurs, especially for fungi, since bacteria are generally more resistant to fire than fungi [88,89]. In addition, fire decreases interspecific competition due to a loss in vegetation, meaning that *A. longifolia* becomes an available host able to control the symbiont’s recruitment, which also possibly explains an increased number of infected cells. Indeed, for the bacteriome, a similar diversity was found, which was reflected in the top 10, which had a common top 3, as mentioned above. In this sense, *A. longifolia* seems to be able to maintain and recruit bacterial partners easily and steadily (as also shown by the lower dissimilarity found), reinforcing the importance of nodulation in post-fire recovery, particularly considering the potential role of bacteria in N fixation. Regarding mycobiome, the decrease in diversity can be due to a loss of fungal richness and mycorrhizal colonization after fire, as pointed in the recent studies of Dove and Hart [90]. Our study shows that *A. longifolia* can recruit a microbiome for root nodules, making it a holobiont [91]. This phytomicrobiome can explain the ease of adaptation, which is an important trait for invasive success, especially after fire, when recruitment is highly dependent on microbial-associated processes [85].

## 5. Conclusions

The structure of the nodules of *A. longifolia* developed in plants grown without and after a fire event was similar, but higher numbers of infected cells and starch granules were present after fire. Through NGS analysis, we identified a microbiome present inside root nodules, including bacteria and fungi, which reinforces the important role of nodulation in the establishment and growth of this invasive plant. These different microbial partners recruited from the surrounding rhizosphere most likely play different ecological roles and the changes found could be related to the ability of the plant to select available partners. The bacteriome seems to change in response to environmental conditions: while N fixation seems to be determinant for sapling establishment after fire, in competitive conditions (unburnt), genera more linked with growth and development were identified. The mycobiome revealed a greater representation of genera commonly described as endophytes in other plants, in various plant organs. For example, some changes occur in the mycobiome after fire occurrence, with *Coniochaeta* becoming highly prevalent and *Penicillium* and *Alternaria* decreasing. Indeed, the presence of fungi inside root nodules can be associated with abiotic stress resistance and/or the facilitation of symbiotic associations. This tripartite symbiosis (i.e., bacteria–fungi–plant), along with the ability to behave as a promiscuous host, could give an advantage to *A. longifolia*, constituting an important trait that can explain its dispersal and invasive success.

## Figures and Tables

**Table 1:** Mean and standard errors (mean ± SE, *n* = 3), number of operational taxonomic units (OTUs), Shannon–Wiener diversity, Pielou evenness, and Bray–Curtis dissimilarity indexes assessed for each microbial group (bacteria and fungi) for each sampling site, unburnt and burnt. Note that Bray–Curtis dissimilarity corresponds to the mean value calculated for each site.

Microbial Group	Site	Classified Reads	OTUs	Shannon-Wiener Index	Pielou Index	Bray-Curtis Dissimilarity
**Bacteria**	Unburnt	478,853	486 ± 90	4.15 ± 0.354	0.670 ± 0.04	0.250
	Burnt	320,485	410 ± 58	4.20 ± 0.187	0.651 ± 0.02	
**Fungi**	Unburnt	1,054,736	705 ± 133	3.66 ± 0.186	0.518 ± 0.03	0.616
	Burnt	975,557	531 ± 93	2.50 ± 0.656	0.363 ± 0.09	

## Data Availability

Further data and details are provided in the Supplementary Material file. The full dataset used in this study is available upon request to the corresponding author.

## Supplementary Materials

The following supporting information can be downloaded at: https://www.mdpi.com/article/10.3390/biology12091168/s1, Figure S1: Squematic drawing of the sampling area including burnt area and the three unburnt and burnt sites.; Figure S2: Rarefaction curves using the observed operational taxonomic units (OTUs) (a and c) and Shannon-Winner index (b, d) from the three unburnt (UB) and three burnt (B) sites, for bacteria (a, b) and fungi (c, d); Figure S3: Beta diversity using Bray-Curtis dissimilarity of bacterial (a) and fungal (b) communities in each site considering the three plots using the observed operational taxonomic units (OTUs) abundance.
