# Supplementary material for: What If Root Nodules Are a Guesthouse for a Microbiome? The Case Study of Acacia longifolia
Source: Biology (Basel). 2023 Aug 24;12(9):1168. doi: 10.3390/biology12091168 (PMC10525506; doi:10.3390/biology12091168)
Supplement: Supplementary file 1 [file biology-12-01168-s001.zip › biology-2517697-supplementary.pdf]

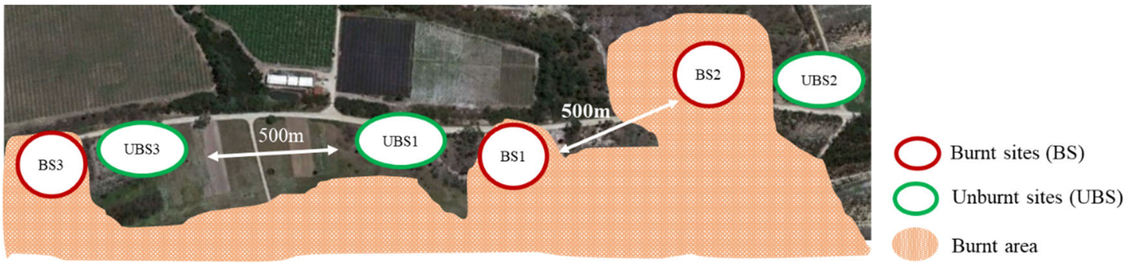

Figure S1. Squematic drawing of the sampling area including burnt area and the three unburnt and burnt sites.

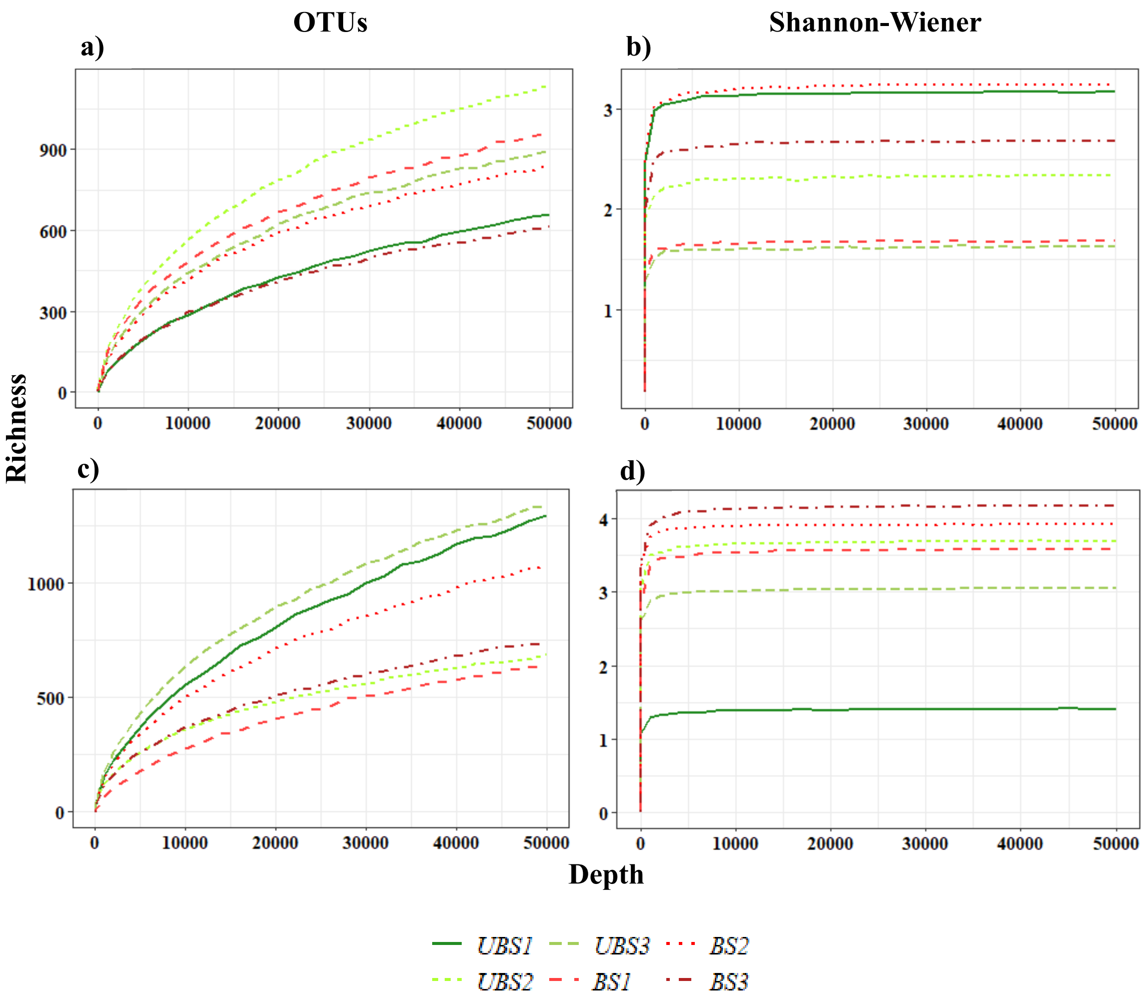

Figure S2. Rarefaction curves using the observed operational taxonomic units (OTUs) (a and c) and Shannon-Winner index (b, d) from the three unburnt (UB) and three burnt (B) sites, for bacteria (a, b) and fungi (c, d).

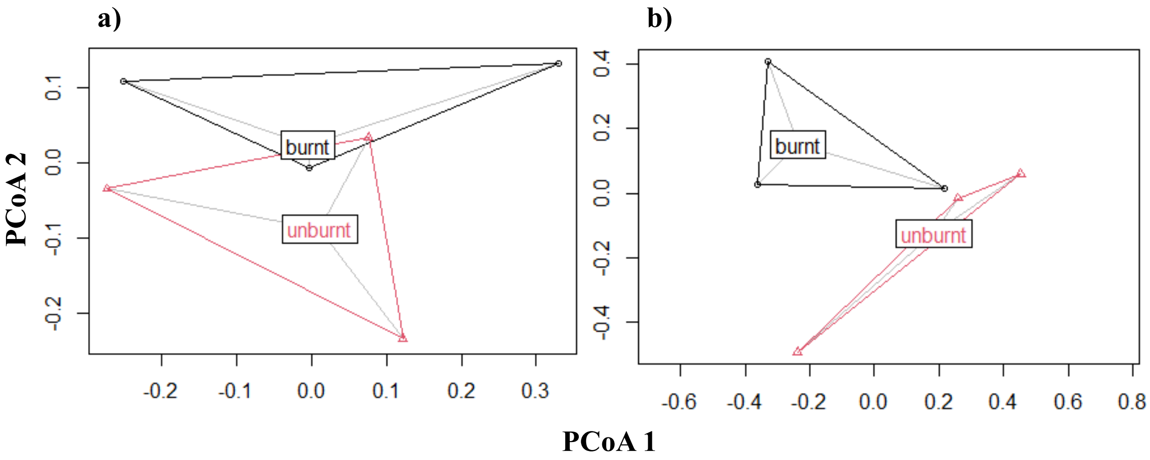

Figure S3. Beta diversity using Bray-Curtis dissimilarity of bacterial (a) and fungal (b) communities in each site considering the three plots using the observed operational taxonomic units (OTUs) abundance.
